# CD26/dipeptidyl peptidase IV enhances expression of topoisomerase II alpha and sensitivity to apoptosis induced by topoisomerase II inhibitors

**DOI:** 10.1038/sj.bjc.6601253

**Published:** 2003-09-30

**Authors:** K Sato, U Aytac, T Yamochi, T Yamochi, K Ohnuma, K S McKee, C Morimoto, N H Dang

**Affiliations:** 1 Department of Lymphoma/Myeloma, MD Anderson Cancer Center, 1515 Holcombe Boulevard, Houston, TX 77030, USA; 2 Department of Clinical Immunology and AIDS Research Center, Institute of Medical Science, University of Tokyo, 4-6-1, Shirokanedai, Minato-ku, Tokyo 108-8639, Japan; 3 Department of Molecular Therapeutics, MD Anderson Cancer Center, 1515 Holcombe Boulevard, Houston, TX 77030, USA

**Keywords:** CD26/DPPIV, apoptosis, topoisomerase II, caspase-9, DNA damage

## Abstract

CD26/dipeptidyl peptidase IV (DPPIV) is a cell surface-bound ectopeptidase with important roles in T-cell activation and tumour biology. We now report that CD26/DPPIV enhances sensitivity to apoptosis induced by the antineoplastic agents doxorubicin and etoposide. In particular, CD26/DPPIV presence is associated with increased susceptibility to the mitochondrial pathway of apoptosis, documented by enhanced cleavage of poly (ADP ribose) polymerase (PARP), caspase-3 and caspase-9, Bcl-xl, and Apaf-1, as well as increased expression of death receptor 5 (DR5). We also show that the caspase-9-specific inhibitor z-LEHD-fmk inhibits drug-mediated apoptosis, leading to decreased PARP and caspase-3 cleavage, and reduced DR5 expression. Importantly, through detailed studies that demonstrate the association between topoisomerase II alpha expression and DPPIV activity, our data provide further evidence of the key role played by CD26 in biological processes.

CD26 is a 110-kDa type II cell surface glycoprotein with diverse functional properties that is widely expressed on various tissues (Dang and Morimoto, 2002; Sato and Dang, 2003). Its extracellular domain encodes a membrane-associated dipeptidyl peptidase IV (DPPIV) activity capable of processing amino-terminal dipeptides from polypeptides with either L-proline or L-alanine in the penultimate position (Oravecz *et al*, 1997). In addition, CD26 acts as an alternative pathway of T-cell activation through its physical and functional association with molecules involved in T-cell signal transduction processes, including CD45, mannose 6-phosphate/insulin-like growth factor II receptor and adenosine deaminase (ADA) (Morimoto *et al*, 1989; Dang *et al*, 1990a, 1990b, 1990c, 1990d, 1991; Ishii *et al*, 2001; Dang and Morimoto, 2002; Sato and Dang, 2003). With its deficiency being a cause of severe combined immune deficiency, ADA plays a key role in the development and function of lymphoid tissues and by regulating surface expression of ADA, CD26 potentially has an important role in adenosine metabolism and immune regulation (Sato and Dang, 2003). Meanwhile, recent findings suggest that CD26 has a role in the development of certain types of neoplasms (Morrison *et al*, 1993; Sedo and Revoltella, 1995; Tanaka *et al*, 1995; Stecca *et al*, 1997). B-chronic lymphocytic leukaemia cells have high levels of CD26 protein expression and mRNA transcripts (Bauvois *et al*, 1999); whereas the more aggressive T-cell malignancies, such as T-cell acute lymphoblastic leukaemia or T-cell CD30+ anaplastic large-cell lymphoma, express higher CD26 level as compared to the more indolent T-cell diseases like mycosis fungoides (Carbone *et al*, 1995; Jones *et al*, 2001).

Topoisomerase II inhibitors are widely used agents in cancer treatment (Froelich-Ammon and Osheroff, 1995), exploiting the catalytic activity of topoisomerase II alpha by increasing permanent DNA damage (Beck *et al*, 1999). Previous findings have shown that DNA damage mediated by topoisomerase II inhibitors induces apoptosis (Beck *et al*, 1999; Mow *et al*, 2001), particularly through cytochrome *c* release (Liu *et al*, 1996; Kluck *et al*, 1997), Apaf-1 involvement (Perkins *et al*, 2000; Lauber *et al*, 2001), and subsequent caspase-9 activation (Mow *et al*, 2001). We have previously shown that surface expression of CD26/DPPIV enhances sensitivity of CD26 Jurkat T-cell transfectants to G2/M arrest mediated by the topoisomerase II inhibitors doxorubicin and etoposide (Aytac *et al*, 2001, 2003). Expanding on these previous findings, we now examine the effect of CD26/DPPIV expression on apoptosis induced by doxorubicin and etoposide. In this paper, we demonstrate that CD26/DPPIV presence enhances sensitivity to apoptosis induced by topoisomerase II inhibitors, associated with increased cleavage of Bcl-xl, Apaf-1, procaspase-9, procaspase-3, and PARP, as well as increased expression of DR5. Meanwhile, pretreatment with the caspase-9-specific inhibitor z-LEHD-fmk significantly reduces etoposide-mediated apoptotic events. Importantly, we present detailed data from different experimental approaches demonstrating the association between DPPIV enzyme activity and topoisomerase II alpha expression. Our findings thus emphasise the increasingly important role of the multifaceted CD26/DPPIV molecule in biological processes, while the functional association between CD26/DPPIV and topoisomerase II alpha may be exploited for future treatments of selected cancers.

## MATERIALS AND METHODS

### Cells and reagents

Human CD26 Jurkat T-cell leukaemia stable transfectants have been described and characterised previously regarding CD26 surface expression and associated DPPIV enzyme activity (Aytac *et al*, 2001, 2003). The Jurkat cell lines include: (a) wild-type CD26-transfected Jurkat cell lines (wtCD26); (b) Jurkat cell lines transfected with mutant CD26 containing an alanine at the putative catalytic serine residue at position 630, resulting in a mutant CD26-positive/DPPIV-negative Jurkat transfectant (S630A); (c) Jurkat cell lines transfected with mutant CD26 containing point mutations at ADA-binding site residues 340–343, with amino acid L340, V341, A342, and R343 being replaced by amino acids P340, S341, E342, and Q343, resulting in a mutant CD26-positive/DPPIV-positive Jurkat transfectant incapable of binding ADA (340–344); and (d) nontransfected control Jurkat cells (parental). Jurkat transfectants were maintained in culture media, which consisted of RPMI 1640 supplemented with 10% FCS, penicillin (100 U ml^−1^), streptomycin (100 *μ*g ml^−1^), and G418 (0.25 mg ml^−1^; Life Technologies Inc., NY, USA) Nontransfectant control Jurkat cells were maintained in the same culture media without G418. Annexin V- fluorescein isothiocyanate (FITC) was from BD PharMingen, CA, USA. Anti-PARP, cytochrome *c*, and caspase-3 Abs were from BD Phar-Mingen; anti-actin was from Sigma Chemical Co, MO, USA; anti-caspase-9 was from Cayman, MI, USA. Anti-DR5 Abs were from Cayman. Anti-Bcl-xl and Apaf-1 Abs were from BD Transduction Laboratories, CA, USA. Anti-topoisomerase II alpha was from Roche, IN, USA. Caspase-9 inhibitor (z-LEHD-fmk) was from BD PharMingen. Diisopropyl fluorophosphate (DFP) was obtained from SIGMA, MO, USA. Substrate for DPPIV, Gly-Pro-*p*-nitroanilide-tosylate (GPNT), was purchased from WAKO, Japan. Etoposide was purchased from SIGMA and was dissolved in sterile DMSO. Doxorubicin was purchased from Calbiochem, CA, USA and was dissolved in sterile PBS. Soluble CD26 molecules were produced by Chinese hamster ovary cells and purified as described previously (Tanaka *et al*, 1994).

### Annexin/propidium iodide (PI) assays

Exposure of phosphatidylserine residues was quantified by surface Annexin V staining as previously described (Raynal and Pollard, 1994). Briefly, cells were washed in binding buffer (10 mM HEPES, pH 7.4, 2.5 mM CaCl_2_, 140 mM NaCl), resuspended in 100 *μ*l and incubated with 0.5 *μ*l ml^−1^ annexin V-FITC and 2.5 *μ*g ml^−1^ PI for 15 min in the dark. Cells were then washed again and resuspended in 400 *μ*l of binding buffer, then flow cytometric analysis (FACScan; Becton Dickinson, CA, USA) was performed. A total of 10 000 cells were acquired per sample and data were analysed using Cellquest software (Becton Dickinson).

### SDS–PAGE and immunoblotting

After incubation at 37°C in culture media and etoposide or doxorubicin at the concentrations and duration indicated, cells were harvested from wells, washed with PBS, and lysed in lysis buffer consisting of 1% NP-40, 0.5%, deoxycolate, 0.1% SDS, 1 mM phenylmethylsulphonyl fluoride, 1 mM benzamidine, 10 *μ*g ml^−1^ aprotinin, 50 *μ*g ml^−1^ leupeptin, 10 *μ*g ml^−1^ soybean trypsin inhibitor and 1 *μ*g ml^−1^ pepstatin. After incubating on ice for 5 min, nuclei were removed by centrifugation and supernatants were collected as whole-cell lysates. Sample buffer (4 ×) consisting of 20% glycerol, 4.6% SDS, 0.5 M Tris (pH 6.8), 4% *β*-mercaptoethanol, and 0.2% bromophenol blue was added to the appropriate aliquots of supernatants. After boiling, protein samples were submitted to SDS–PAGE analysis on an 8% gel under standard conditions using a mini-Protean II system (Bio-Rad, CA, USA). For immunoblotting, the proteins were transferred onto nitrocellulose (Immobilon-P; Millipore, MA, USA). After overnight blocking at 4°C in blocking solution consisting of 0.1% Tween 20 and 5% BSA in Tris-buffered saline, membranes were blotted with the appropriate primary antibodies diluted in blocking solution for 1 h at room temperature. Membranes were then washed with blocking solution, and appropriate secondary antibodies diluted in blocking solution were then applied for 1 h at room temperature. Secondary antibodies were goat anti-mouse or goat anti-rabbit horseradish peroxidase conjugates (Dako). Membranes were then washed with blocking solution, and proteins were subsequently detected by chemiluminescence (Amersham Pharmacia Biotech, NJ, USA).

### Dipeptidyl Peptidase IV enzyme activity assays

As previously described (Kajiyama *et al*, 2002), DPPIV enzyme activity was measured spectrophotometrically using GPNT, a substrate for DPPIV. A 1 × PBS-washed whole-cell suspension was prepared and 5 × 10^5^ cells were resuspended in 200 *μ*l of PBS into 96-well plate, then GPNT was added at a final concentration of 0.24 mM. The absorption was measured at 405 nm using microplate spectrophotometer (BIO-TEK Instruments, inc., VE, USA) twice, just before the addition of the substrate and after 60 min incubation at 37°C. Dipeplidyl peptidase enzyme activity was calculated from the increase of absorption between 0 and 60 min.

### Inhibition of DPPIV enzyme activity

As described previously (Koreeda *et al*, 2001; Kajiyama *et al*, 2002), DFP was used as the DPPIV chemical inhibitor for inhibition assays. To evaluate effect of continuous exposure to DFP, wtCD26 transfectants or parental Jurkat cells were incubated in culture media alone (DFP−), culture media containing 100 *μ*M DFP for 2 h or for 6 h (DFP+). A representative sample of cells reflecting each treatment condition was obtained for DPPIV enzyme activity assays or to examine topoisomerase II alpha expression. Alternatively, wtCD26 Jurkat transfectants were incubated in culture media; or in culture media with 100 *μ*M DFP for 4 h; or they were incubated in culture media with 100 *μ*M DFP for 4 h, then washed twice in PBS to ensure removal of DFP followed by incubation in culture media for 2 or 8 h. A representative sample of cells reflecting each treatment condition was obtained for DPPIV enzyme activity assays or to examine topoisomerase II alpha expression. For all treatment conditions, trypan blue uptake assays consistently showed >90% cell viability (data not shown).

### Preparation of cytosol fractions

As previously described (Haridas *et al*, 2001; Nishimura *et al*, 2001), Jurkat cells (4.0 × 10^7^) were suspended in 1 ml sucrose buffer (250 mM sucrose in 30 mM Tris HCl, pH 7.4) and transferred into an N_2_ cavitation chamber (PARR Instruments, Moline, IL, USA). The cells were subjected to N_2_ cavitation (250 psi for 5 min) according to the manufacturer's instructions. Under these conditions, most of the cell membrane was disrupted with no change in the mitochondrial respiratory activity. Next, DNA and the nuclear fraction were removed by centrifugation (1500 **g** for 2 min). The supernatant was further centrifuged (16 000 **g** for 10 min), and the supernatant was used as the cytosol fraction.

### Preparation of nuclear extracts

Cells (10 × 10^6^) were harvested and allowed to swell for 15 min on ice in cytoplasmic extraction buffer (10 mM HEPES, 10 mM KCl, 0.1 mM EDTA, 0.1 mM EGTA, 1 mM DTT, 1 mM PMSF, 2 *μ*g ml^−1^ leupeptin, 2 *μ*g ml^−1^aprotinin, and 0.5 mg ml^−1^ benzamidine). Then NP-40 (final concentration 0.3%) was added into that cell suspension and vortexed for 10 s. After 2 min-centrifugation at 16 000 **g**, the supernatant was discarded. The pellet was then incubated with nuclear extraction buffer (20 mM HEPES, 400 mM KCl, 1 mM EDTA, 1 mM EGTA, 1 mM DTT, 0.5 mM PMSF, 2 *μ*g ml^−1^ leupeptin, 2 *μ*g ml^−1^ aprotinin, and 0.5 mg ml^−1^ benzamidine) for 30 min on ice with intermittent vortexing. The suspension was centrifuged at 16000 **g** for 6 min, and the supernatant was saved as the nuclear extract.

## RESULTS

### Effect of CD26/DPPIV expression on apoptosis of Jurkat cells mediated by topoisomerase II inhibitors

Annexin V/PI assays show that wtCD26 Jurkat transfectants are more sensitive to the apoptotic effect of etoposide than S630A or parental control Jurkat cells. Meanwhile, 340–4 transfectants (340–4) exhibit higher level of drug-induced apoptosis, similar to that of wtCD26 transfectants (Figure 1A

**Figure 1:**
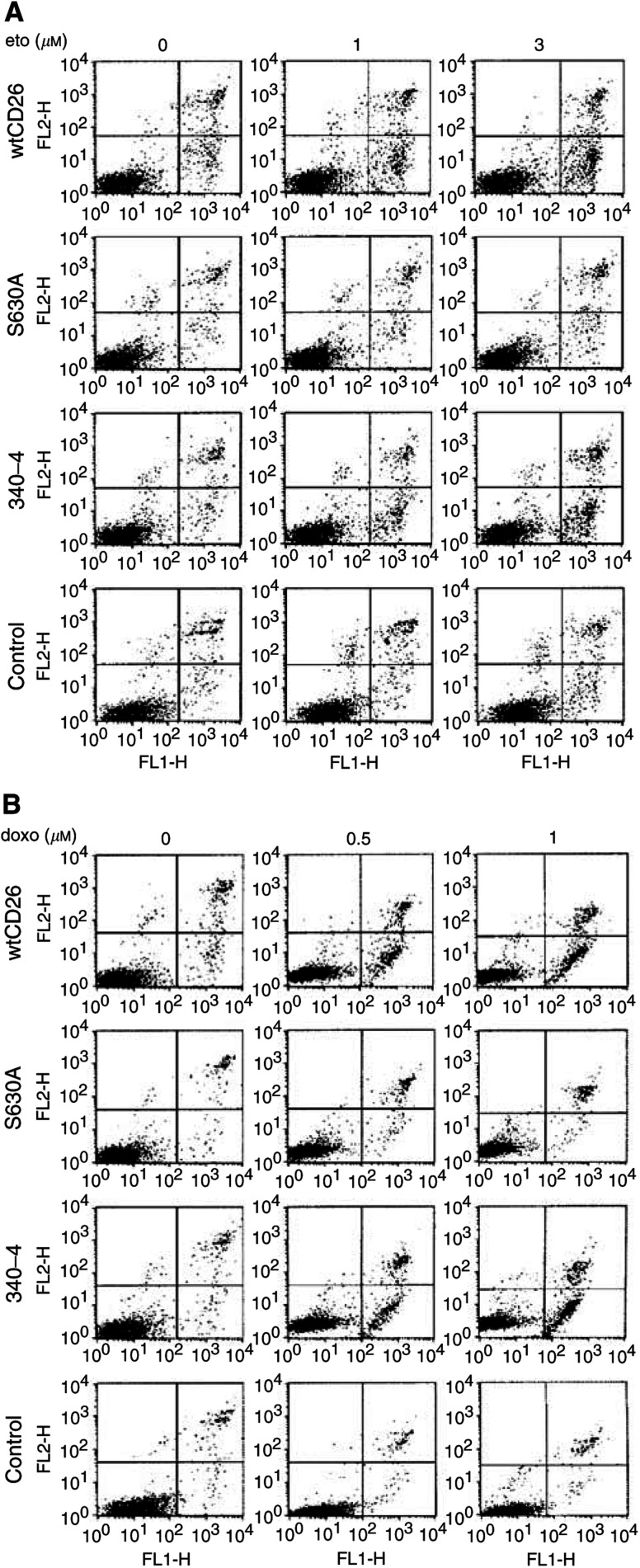
Enhancing effect of CD26/DPPIV surface expression on apoptosis induced by topoisomerase II inhibitors. CD26 Jurkat transfectants were incubated at 37°C in culture media alone or culture media containing etoposide (**A**) for 14 h or doxorubicin (**B**) for 16 h at the concentrations indicated. Cells were then harvested and Annexin V/PI assays were performed as described in Materials and Methods. *wtCD26*: wild-type CD26 Jurkat transfectant; *S630A*: Jurkat cells transfected with mutant CD26 containing an alanine at the putative catalytic serine residue at position 630, resulting in a mutant CD26-positive/DPPIV-negative Jurkat transfectant; *control*: nontransfected parental Jurkat; *340–4*: Jurkat cells transfected with mutant CD26 containing point mutations at the ADA-binding site residues 340–343, with amino acids L_340_, V_341_, A_342_, and R_343_ being replaced by amino acids P_340_, S_341_, E_342_, and Q_343_, resulting in a mutant CD26-positive/DPPIV-positive mutant CD26 Jurkat transfectant incapable of binding ADA. Data are representative of three separate experiments. (**C**) wtCD26 Jurkat transfectants and parental cells were treated with doxorubicin over the indicated time intervals and drug concentrations. a: 12 h, b: 24 h, c: 36 h. Data are representative of three separate experiments.

). Furthermore, wtCD26 and 340–4 cells display greater apoptosis when treated with doxorubicin as compared with parental or S630A Jurkat cells (Figure 1B). Time course studies to evaluate the effect of doxorubicin on apoptosis in these CD26 Jurkat transfectants are also performed. Our data show that wtCD26 Jurkat transfectants consistently exhibit greater drug-induced apoptosis over the time intervals and drug concentrations tested than parental Jurkat (Figure 1C

**Figure 1:**
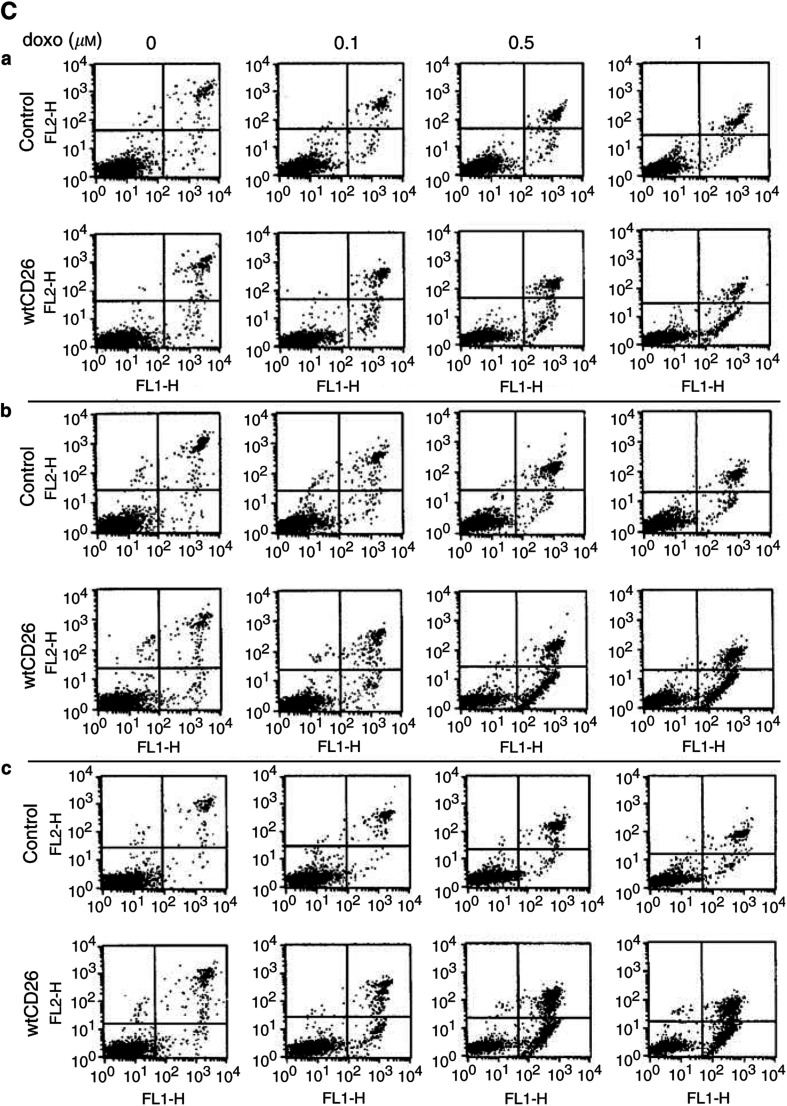

), strongly suggesting that the presence of CD26 directly enhances cellular sensitivity to topoisomerase II inhibitor-induced apoptosis. Similar results are obtained with etoposide treatment (data not shown). Similarly, both the wtCD26 and 340–4 Jurkat transfectants are more sensitive to etoposide-mediated PARP cleavage (Figure 2

**Figure 2:**
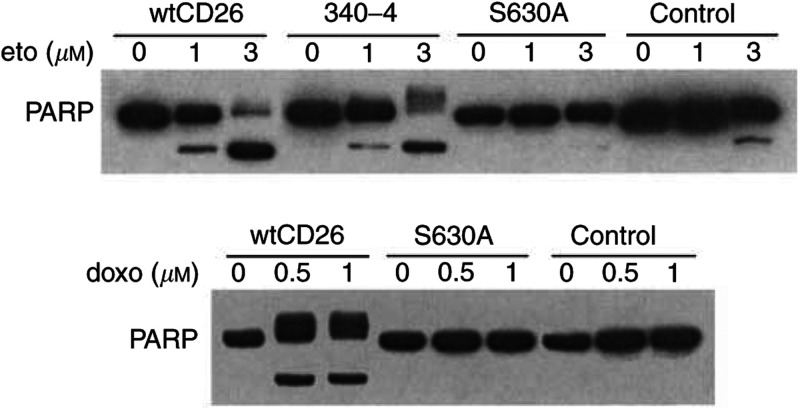
CD26/DPPIV-associated enhancement in PARP cleavage induced by topoisomerase II inhibitors. CD26 Jurkat transfectants were incubated at 37°C with media containing etoposide for 16 h or doxorubicin for 18 h at the indicated doses. Cells were then harvested, and whole-cell lysates were obtained. Following SDS–PAGE of lysates, immunoblotting studies for PARP and *β*-actin were performed as described in Materials and Methods. The cleaved product of PARP was detected at ∼85 kDa. Each lane was loaded with 30 *μ*g of protein.

), as compared with parental cells and S630A transfectants. Similar results are seen when cells are treated with doxorubicin. These data hence suggest that the presence of CD26, especially its associated DPPIV enzymatic activity, enhances apoptosis mediated by topoisomerase II inhibitors.

### Effect of CD26/DPPIV surface expression on the mitochondrial pathway of apoptosis induced by etoposide

**Figure 3:**
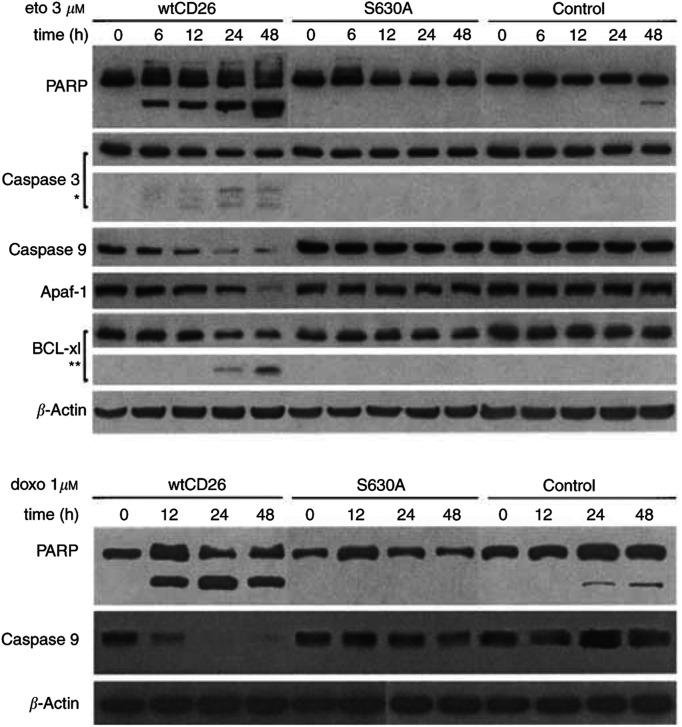
Time course study of the effect of CD26/DPPIV surface expression on etoposide-induced apoptosis. Jurkat cells were incubated at 37°C with media containing 3 *μ*M etoposide or 1 *μ*M doxorubicin for the indicated time periods at the indicated doses. Cells were then harvested, and cytosol fractions were obtained as described in Materials and Methods. Following SDS–PAGE of lysates, immunoblotting studies with specific antibodies for PARP, caspase-9, caspase-3, Apaf-1, Bcl-xl, and *β*-actin were performed as described in Materials and methods (^*^): caspase-3 cleaved products; (^**^): Bcl-xl cleaved products. Each lane was loaded with 30 *μ*g of protein.

Previous work demonstrated that DNA damage mediated by topoisomerase II inhibitors induces apoptosis through the mitochondrial pathway. Our time course analyses (Figure 3

) show that etoposide treatment results in enhanced cleavage of PARP and procaspase-3, leading to increased levels of the cleaved 17 kDa caspase-3 bands in wtCD26 transfectants, as compared with S630A and parental Jurkat cells. Moreover, our work shows that etoposide treatment leads to significantly greater cleavage of procaspase-9 in wtCD26 cells compared to S630A and parental control Jurkat. We also demonstrate greater cleavage of the 130 kDa proform of Apaf-1 in etoposide-treated wtCD26 Jurkat transfectants as compared with parental control or S630A Jurkat. Furthermore, the increase in sensitivity to etoposide-induced apoptosis in wtCD26 transfectants is accompanied by greater cleavage of the full-length antiapoptotic molecule Bcl-xl (Fujita *et al*, 1998) and a resultant rise in the 18 kDa cleaved band. Taken together, our results indicate that CD26/DPPIV enhances etoposide-mediated apoptosis of Jurkat cells by affecting cellular processes known to be involved in drug-mediated apoptosis, including those involving caspase-9 processing and the mitochondrial pathway, as well as processing of bcl-2-related molecules. Similarly, time course analyses demonstrate that wtCD26 Jurkat transfectants exhibit greater apoptosis through caspase-9 processing when treated with doxorubicin, as demonstrated by greater cleavage of PARP and procaspase-9.

### Effect of the caspase-9 inhibitor z-LEHD-fmk on etoposide-induced apoptosis in CD26 Jurkat transfectants

**Figure 4:**
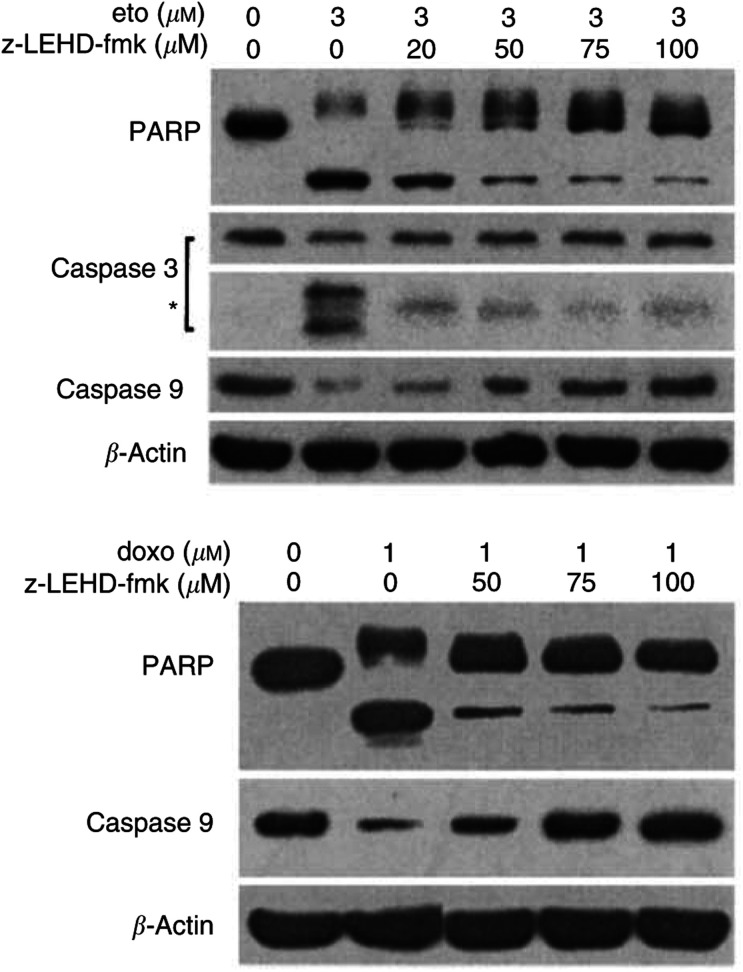
Effect of caspase-9 inhibitor z-LEHD-fmk on etoposide-induced apoptosis in wtCD26 Jurkat transfectant. wtCD26 Jurkat transfectants were incubated at 37°C for 2 h of preincubation with z-LEHD-fmk at varying doses, and then treated with 3 *μ*M etoposide or 1 *μ*M doxorubicin for 16 h. Cells were then harvested, and whole-cell lysates were obtained as described in Materials and Methods. Following SDS–PAGE of lysates, immunoblotting studies for PARP, caspase-3, caspase-9, and *β*-actin were performed as described in Materials and Methods (^*^): caspase-3 cleaved products. Each lane was loaded with 30 *μ*g of protein.

To further confirm our findings that CD26 affects etoposide-induced apoptosis through caspase-9-related events, we evaluated the effect of the caspase-9 inhibitor z-LEHD-fmk on this process. Western blot analyses show that pretreatment with z-LEHD-fmk significantly abrogates the effect of etoposide on wtCD26 Jurkat transfectants. As shown in Figure 4

, etoposide-mediated cleavage of procaspase-9 is inhibited by z-LEHD-fmk in a dose-dependent manner. Furthermore, cleavage of procaspase-3 and PARP, events downstream of caspase-9 processing, is significantly reduced following pretreatment with the caspase-9 inhibitor. Our data therefore indicate CD26 augments etoposide-induced apoptosis in CD26 Jurkat transfectants through caspase-9-related events. Similarly, pretreatment with z-LEHD-fmk significantly decreases the effect of doxorubicin on wtCD26 Jurkat transfectants, as measured by cleavage of PARP and procaspase-9.

### Effect of the DPPIV enzyme inhibitor DFP on topoisomerase II alpha expression

**Figure 5:**
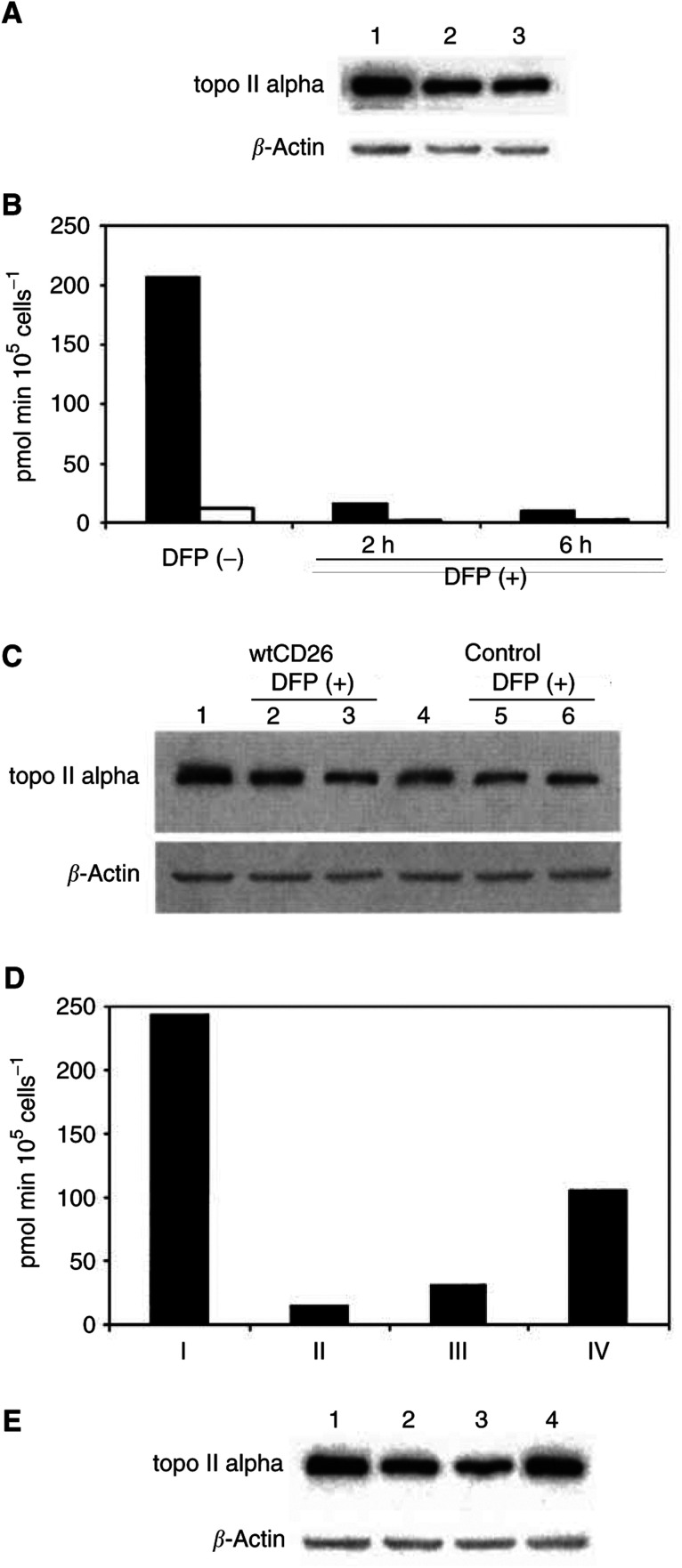
Effect of inhibition of DPPIV activity on topoisomerase II alpha expression. (**A**) After incubation of Jurkat cells at 37°C for 24 h in culture media, cells were harvested and nuclear extracts were obtained. Following SDS–PAGE of lysates, immunoblotting studies were performed for topoisomerase II alpha or *β*-actin as described in Materials and Methods. Each lane was loaded with 30 *μ*g of protein. Lane 1: wtCD26 Jurkat transfectant, lane 2: S630A mutant transfectant, lane 3: parental Jurkat. (**B**) wtCD26 Jurkat transfectants or parental Jurkat were incubated in culture media alone (DFP−), culture media containing 100 *μ*M DFP for 2 or 6 h (DFP+). A representative sample of cells reflecting each treatment condition was obtained, and DPPIV enzyme activity assays were then performed as described in Materials and Methods. (**C**) wtCD26 Jurkat transfectants (lanes 1–3) or parental Jurkat (lanes 4–6) were incubated in culture media alone (lanes 1, 3), culture media containing 100 *μ*M DFP for 2 h (lanes 2, 5) or for 6 h (lanes 3, 6). Cells were harvested, and nuclear extracts were obtained. Following SDS–PAGE of lysates, immunoblotting studies for topoisomerase II alpha or *β*-actin were performed as described in Materials and Methods. Each lane was loaded with 30 *μ*g of protein. (**D**) wtCD26 Jurkat transfectants were incubated in culture media (bar I), or in culture media with 100 *μ*M DFP for 4 h (bar II), or they were incubated in culture media with 100 *μ*M DFP for 4 h, then washed twice in PBS to ensure removal of DFP followed by incubation in culture media for 2 h (bar III) or 8 h (bar IV). A representative sample of cells reflecting each treatment condition was obtained, and DPPIV enzyme activity assays were then performed as described in Materials and Methods. (**E**) wtCD26 Jurkat transfectants were incubated in culture media (lane 1), or in culture media with 100 *μ*M DFP for 4 h (lane 2), or they were incubated in culture media with 100 *μ*M DFP for 4 h, then washed twice in PBS to ensure removal of DFP followed by incubation in culture media for 2 h (lane 3) or 8 h (lane 4). Cells were then harvested and nuclear extracts were obtained. Following SDS–PAGE of lysates, immunoblotting studies for topoisomerase II alpha or *β*-actin were performed as described in Materials and Methods. Each lane was loaded with 30 *μ*g of protein.

We previously showed that topoisomerase II alpha expression and catalytic activity are higher in wtCD26 Jurkat transfectant than S630A or parental cells (Aytac *et al*, 2003) (Figure 5A

). To further evaluate in detail the effect of DPPIV activity on topoisomerase II alpha expression, we examined the effect of the DPPIV chemical inhibitor DFP (Koreeda *et al*, 2001; Kajiyama *et al*, 2002) on topoisomerase II alpha expression. Continuous treatment with DFP results in inhibition of DPPIV enzyme activity in wtCD26 Jurkat transfectants (Figure 5B), associated with decreased expression of topoisomerase II alpha in these cells (Figure 5C). On the other hand, expression of topoisomerase II alpha in parental Jurkat cells is not significantly affected by continuous exposure to DFP, as expected. Additionally, we examined the status of DPPIV enzyme activity and topoisomerase II alpha expression following DFP treatment. For this purpose, following treatment with DFP, wtCD26 cells were washed and incubated in culture media for the indicated time periods. We demonstrate that recovery of DPPIV enzyme activity is associated with recovery of topoisomerase II expression (Figure 5D and E). These results further corroborate and expand on our earlier findings regarding the importance of DPPIV enzyme activity in topoisomerase II alpha expression in CD26 Jurkat transfectants.

### Effect of soluble CD26 molecules on topoisomerase II alpha expression and sensitivity to doxorubicin

**Figure 6:**
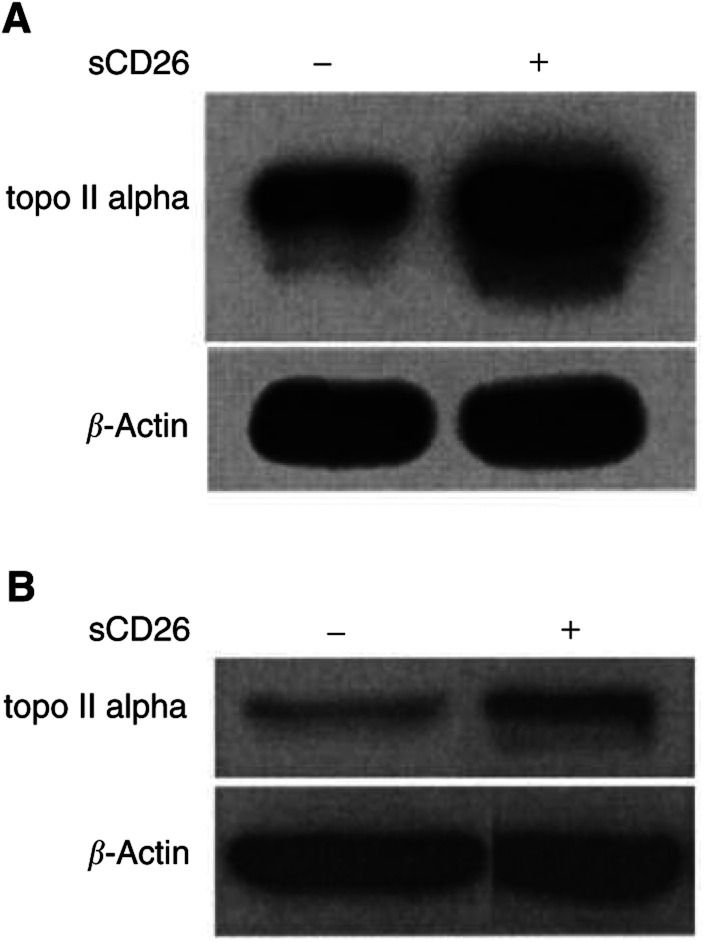
Effect of soluble CD26 molecules on topoisomerase II alpha expression. Parental Jurkat cells (**A**) or Jiyoye cells (**B**) were incubated overnight in culture media alone (−) or culture media containing soluble CD26 (sCD26) molecules (300 *μ*g ml^−1^) (+) at 37°C. Cells were then harvested and nuclear extracts were obtained. Following SDS–PAGE of lysates, immunoblotting studies for topoisomerase II alpha or *β*-actin were performed as described in Materials and Methods. Each lane was loaded with 30 *μ*g of protein.

**Figure 7:**
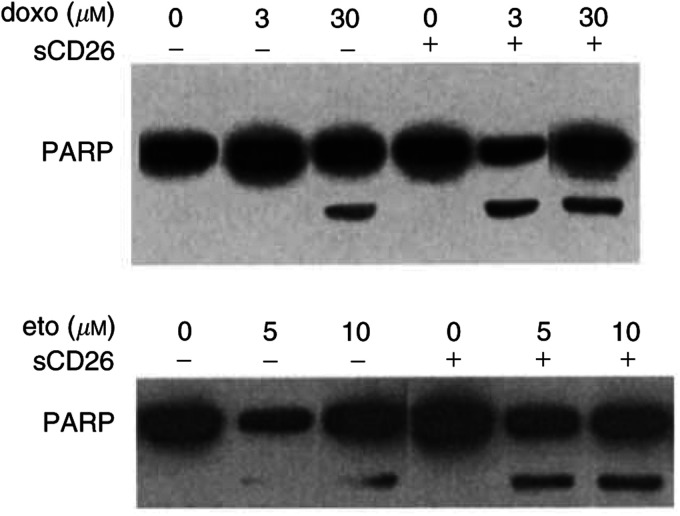
CD26-associated enhancement of doxorubicin or etoposide-induced PARP cleavage. Parental Jurkat cells were incubated overnight in culture media alone (−) or culture media containing soluble CD26 (sCD26) molecules (300 *μ*g ml^−1^) (+) at 37°C, followed by incubation with doxorubicin or etoposide at the indicated concentrations for 16 h. Cells were then harvested, and whole-cell lysates were obtained as described in Materials and Methods. Following SDS–PAGE of lysates, immunoblotting studies for PARP or *β*-actin were performed as described in Materials and Methods. Each lane was loaded with 30 *μ*g of protein.

It is theoretically possible that DPPIV effect is dependent on surface expression of the intact CD26/DPPIV molecule. To address this issue, we evaluate the effect of soluble CD26 (sCD26) molecules on topoisomerase II alpha expression. As shown in Figure 6

, incubation with sCD26 molecules results in a significant increase in topoisomerase II alpha protein expression in parental control Jurkat cells (A) or Jiyoye cells (B). Along with an increase in topoisomerase II alpha expression, incubation of parental Jurkat cells with sCD26 molecules also results in enhanced doroxubicin-induced or etoposide-induced PARP cleavage (Figure 7

). Our findings further confirm that the presence of DPPIV activity itself, and not necessarily surface expression of CD26/DPPIV, augments topoisomerase II alpha expression, leading to a resultant increase in sensitivity to topoisomerase II inhibitors.

### Caspase-9-dependent involvement of DR5 in etoposide-induced apoptosis in CD26 Jurkat transfectants

**Figure 8:**
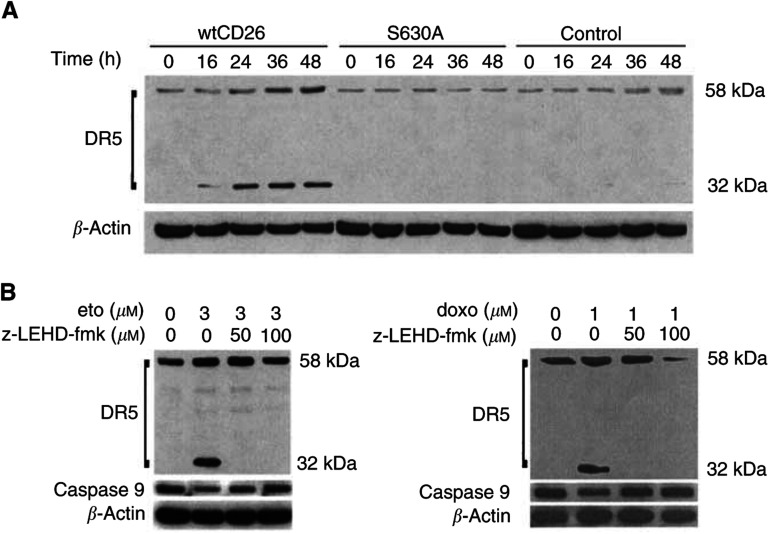
Effect of CD26/DPPIV on DR5 expression induced by etoposide treatment. (**A**) Jurkat cells were incubated at 37°C in culture media containing etoposide (3 *μ*M) for the indicated time periods at the indicated doses. Cells were then harvested, and whole-cell lysates were obtained as described in Materials and Methods. Following SDS–PAGE of lysates, immunoblotting studies for DR5 and *β*-actin were performed as described in Materials and Methods. Each lane was loaded with 30 *μ*g of protein. Anti- DR5 mAb detects two bands of 58 and 32 kDa. (**B**) Following 2 h of preincubation at 37°C with varying doses of z-LEHD-fmk, wtCD26 Jurkat transfectants were treated with 3 *μ*M etoposide or 1 *μ*M doxorubicin for 48 h. Cells were then harvested, and whole cell lysates were obtained as described in Materials and Methods. Following SDS–PAGE of lysates, immunoblotting studies for DR5, caspase-9, and *β*-actin were performed as described in Materials and Methods. Each lane was loaded with 30 *μ*g of protein.

Expression of death receptor 5 (DR5), a member of the TRAIL (tumour necrosis factor-related apoptosis-inducing ligand) family, is upregulated following treatment with such DNA-damaging agents as doxorubicin and etoposide (Gibson *et al*, 2000). We now demonstrate that etoposide treatment leads to a greater increase in the levels of the 58 kDa DR5 in wtCD26 transfectants as compared with S630A or parental Jurkat cells (Figure 8A

). Interestingly, Western blotting analyses with anti-DR5 mAb also detect the expression of a smaller 32 kDa band with etoposide treatment, with its levels again being significantly higher in wtCD26 Jurkat than S630A or parental cells. In time course studies, the appearance of the 32 kDa band consistently precedes the observed increase in expression levels of the 58 kDa band.

We have already demonstrated that etoposide-induced apoptosis in wtCD26 Jurkat transfectant involves caspase-9 processing. To determine whether the enhancement in DR5 expression in etoposide-treated wtCD26 Jurkat is dependent on caspase-9-related events, we examined DR5 expression in cells treated with etoposide following preincubation with the caspase-9-specific inhibitor z-LEHD-fmk. As demonstrated in Figure 8B, the increase in the 58 kDa band seen in etoposide-treated wtCD26 cells is significantly attenuated when cells are preincubated with z-LEHD-fmk. In addition, the 32 kDa band induced by etoposide is no longer detectable with z-LEHD-fmk preincubation. Concordant with the findings with etoposide, pretreatment with z-LEHD-fmk similarly inhibits doxorubicin effect on DR5 status.

## DISCUSSION

Through experiments involving CD26 Jurkat transfectants, DPPIV chemical inhibitor and soluble CD26/DPPIV molecules, we provide conclusive evidence that presence of DPPIV enzyme activity results in enhanced topoisomerase II alpha expression, associated with increased sensitivity to apoptosis induced by topoisomerase II inhibitors. Topoisomerase II enzyme plays an important role in the metabolism of DNA topoisomers and is essential for cellular proliferation (Wang, 1996). Two topoisomerase II isoforms, alpha and beta, exist in eukaryotes, coded by two different genes (Drake *et al*, 1989; Goswami *et al*, 1996). In particular, the 170 kDa topoisomerase II alpha isoform is closely associated with the cell cycle, being highly expressed during cellular proliferation, and is the primary target of such topoisomerase II inhibitors as doxorubicin or etoposide (Burden and Osheroff, 1998). These drugs selectively exploit the catalytic activity of topoisomerase II alpha to create DNA damage by increasing the frequency and duration of DNA cleavage sites, causing permanent double-stranded breaks and leading to apoptosis (Froelich-Ammon and Osheroff, 1995; Beck *et al*, 1999; Mow *et al*, 2001). Owing to this mechanism of toxicity, increased enzyme level is associated with enhanced sensitivity, and drug resistance is related with reduced topoisomerase II alpha level (Beck *et al*, 1993, Oloumi *et al*, 2000). Our current findings that the enhanced topoisomerase II alpha expression associated with CD26/DPPIV presence results in greater sensitivity to apoptosis induced by doxorubicin and etoposide are therefore consistent with the known mechanism of action of these topoisomerase II inhibitors.

Etoposide or doxorubicin engages the caspase-9-related mitochondrial pathway of apoptosis (Sun *et al*, 1999). It is also known that perturbation of Bcl-2-related proteins such as Bcl-xl is important for apoptotic processes associated with drug-induced DNA damage, potentially augmenting death signals from the mitochondrial pathway (Liu *et al*, 1996; Kluck *et al*, 1997; Fujita *et al*, 1998). Our results, including those demonstrating increased drug-induced Bcl-xl cleavage associated with CD26/DPPIV expression and the effect of the caspase-9-specific inhibitor z-LEHD-fmk, are consistent with the conclusion that the CD26/DPPIV-associated increase in apoptosis induced by the topoisomerase II inhibitors is mediated through caspase-9 processing and the mitochondrial pathway. Nevertheless, it is theoretically possible that CD26 exerts its influence on cell growth inhibition via other additional pathway(s).

Our results also indicate that CD26/DPPIV expression is associated with enhancement of not only the 58 kDa DR5 protein, but also the smaller 32 kDa form following topoisomerase II inhibitor treatment of Jurkat cells. While being consistent with previous reports demonstrating that DR5 expression is upregulated following treatment with DNA-damaging agents (Gibson *et al*, 2000), our work also demonstrates the existence of the smaller 32 kDa band. While the exact relationship between the 32 kDa band and the full-length 58 kDa band remains to be elucidated, one potential explanation from our work would be that the smaller band represents a precursor form of DR5. Our time course experiments show that while the 32 kDa band is not detected in untreated cells, its appearance in cells treated with etoposide precedes the detectable increase in the expression levels of the 58 kDa band. Additionally, the pretreatment with the caspase-9 inhibitor z-LEHD-fmk completely inhibits topoisomerase II inhibitor-induced expression of the 32 kDa band while abrogating the increased expression of the 58 kDa band. Besides our demonstration of the existence of the 32 kDa band, our work also reveals a functional relationship between caspase-9 and DR5. Previous work has indicated that death signals related with DR5 are subsequently transmitted downstream to caspase-9 processing events (Gibson *et al*, 2000). However, our findings suggest that caspase-9 processing also affects DR5 expression following drug-induced DNA damage, since pretreatment with the caspase-9 inhibitor z-LEHD-fmk negatively affects expression levels of both the 58 and 32 kDa bands in topoisomerase II inhibitor-treated cells.

Previously published work suggested that CD26/DPPIV expression renders human T cells more responsive to activation signals from various stimuli (Dang and Morimoto, 2002). In addition, CD26 expression on selected human tumours are associated with aggressive tumour behaviour (Carbone *et al*, 1995; Sato and Dang, 2003). Our present findings of the association between CD26/DPPIV and topoisomerase II alpha expression may potentially provide an explanation for these previous observations. In view of the role played by topoisomerase II alpha in cellular proliferation, it is possible that the biological behaviour of these CD26-bearing cells reflects in part the higher levels of topoisomerase II alpha. Our present work thus provides additional evidence of the essential role of the multifaceted CD26 molecule in cellular processes. Furthermore, along with our recent study indicating an antitumour effect of anti-CD26 mAb (Ho *et al*, 2001), our findings may provide insights into the design of future novel treatments against selected human tumours based on our knowledge of CD26 biology.
